# An Overview on Industrial and Medical Applications of Bio-Pigments Synthesized by Marine Bacteria

**DOI:** 10.3390/microorganisms9010011

**Published:** 2020-12-22

**Authors:** Ali Nawaz, Rida Chaudhary, Zinnia Shah, Laurent Dufossé, Mireille Fouillaud, Hamid Mukhtar, Ikram ul Haq

**Affiliations:** 1 Institute of Industrial Biotechnology, GC University Lahore, Lahore 54000, Pakistan; ali.nawaz@gcu.edu.pk (A.N.); ridachdry.789@gmail.com (R.C.); syedazinniashah@gmail.com (Z.S.); hamidmukhtar@gcu.edu.pk (H.M.); dr.ikramulhaq@gcu.edu.pk (I.u.H.); 2 CHEMBIOPRO Lab, ESIROI Agroalimentaire, University of Réunion Island, 97400 Saint-Denis, France; mireille.fouillaud@univ-reunion.fr

**Keywords:** natural colors, bio-pigments, quorum sensing, marine bacteria, biosynthesis, biological activities, industrial applications, therapeutic insights, global pigment market

## Abstract

Marine bacterial species contribute to a significant part of the oceanic population, which substantially produces biologically effectual moieties having various medical and industrial applications. The use of marine-derived bacterial pigments displays a snowballing effect in recent times, being natural, environmentally safe, and health beneficial compounds. Although isolating marine bacteria is a strenuous task, these are still a compelling subject for researchers, due to their promising avenues for numerous applications. Marine-derived bacterial pigments serve as valuable products in the food, pharmaceutical, textile, and cosmetic industries due to their beneficial attributes, including anticancer, antimicrobial, antioxidant, and cytotoxic activities. Biodegradability and higher environmental compatibility further strengthen the use of marine bio-pigments over artificially acquired colored molecules. Besides that, hazardous effects associated with the consumption of synthetic colors further substantiated the use of marine dyes as color additives in industries as well. This review sheds light on marine bacterial sources of pigmented compounds along with their industrial applicability and therapeutic insights based on the data available in the literature. It also encompasses the need for introducing bacterial bio-pigments in global pigment industry, highlighting their future potential, aiming to contribute to the worldwide economy.

## 1. Introduction

### 1.1. Microbial Pigments

The production of bio-pigments from bacterial species is being conducted globally with soaring interest under the research of microbial autecology. A massive array of these compounds, also referred to as “bioactive pigmented molecules”, can be derived from both Gram-positive and Gram-negative bacterial species. Production of these pigments in the marine environment is mediated through the complex mechanism of “quorum sensing” [1] or also can be induced through exposure to different stress conditions in external environments. Quorum sensing is the mechanism whereby individual bacterial cells can coordinate with others in their colony to carry out constitutive functions especially involving the secretion of numerous specific chemical compounds. These compounds can help them with survival, competence, bioluminescence, biofilm formation, and even sporulation, etc. Bio-pigments can be produced by triggering regulatory quorum sensing mechanisms of these species and can be extensively used in various bio-medical and bio-industrial sectors, including textiles, food, pharmaceutical, and cosmetic industries, owing to their beneficial attributes and biological activities [2,3]. These are moreover convenient to harvest in large volumes through utilizing simple gene manipulating strategies. The rising consumer concerns regarding safety and quality of industrial products holds a significant ground as to why scientists are shifting their focus towards naturally derived, non-toxic, and eco-friendly pigment alternatives [4].

### 1.2. Bacterial Pigments as Natural Colorants

The use of synthetic pigments goes back to the 1850s when these were put in trend for the first time due to their supercilious coloring properties, lower prices, and easy production strategies [5], the significance of which remains empirically the same to this day. The importance of artificial/synthetic coloring agents is still based on the fact that the appearance of food items influences consumer’s emotions, attitudes, and preferences. Let us say, if a carrot is not red, the consumer is most probably expected to reject it. The same can be applied in regards with the cosmetic industry, where the product apparel decides its fate. Thus, need for “synthetic pigments” cannot be overseen if client orientation is to be fulfilled [6]. The only progress made today is the shift towards naturally derived pigments rather than continuing the use of artificially synthesized ones, which have been denounced for their serious threat to consumer’s well-being [7]. Cancers of skin, liver, and bladder have been found positively related to the use of artificial pigments because of their high azo-dye/heavy metal compositions. Furthermore, the precursors involved and the waste generated through their production process is environmentally hazardous as well [8,9]. The outcry against the use of synthetic colorants in many health-conscious countries has already caused the ban of several artificial colorants, including Blue NO 1, Blue NO 2, Blue FCF, and Yellow NO 6 [10].

Bio-pigments, however, are eco-friendly and proved additionally propitious as antitoxic, antitumor, antioxidant, anticancer, and antimicrobial agents [2]. Other advantages include fast and economic extraction techniques, higher yield, and time- and cost-efficient production. Moreover, the production of microbial pigments can also be made more convenient by the optimization capacity of their growth parameters [11]. Keeping the capacity of bio-pigments into consideration, many biotech industries are now developing protocols for efficient extraction of natural pigments as a replacement to synthetic counterparts. For instance, natural pigments such as zeaxanthin, saproxanthin, myxol and many others which illicit antioxidant activities are being instigated against artificial antioxidants such as butylated hydroxyl toluene and butylated hydroxyl acids [12,13].

### 1.3. Marine Ecosystem as a Source of Pigment Producing Bacterial Species

The study of a likely natural ecosystem serves as the initial-most important research step needed to find an environment that can entertain the diversity of bio-pigment sources. The marine environment is a habitat for almost 80% of all life forms [14]. It serves as a rich source of aquatic microbial species that exhibit comparatively more augmented diversity than their telluric counterparts [15]. The marine environment is presently being considered as an attractive fount for bio-pigment sources [16]. Numerous bacterial isolates from such biotopes have already been tested for pigment production. At the same time, many of them are also being utilized for various industrial purposes as well [15]. The preference of pigments produced by marine microorganisms is based on their ability to persist in extremities such as highly acidic/alkaline environments (pH < 4 and >9), extreme temperatures (−2–15 °C and 60–110 °C), and under limited substrate availability [17,18]. Apart from bacterial isolates, halophilic archaea are extensively disseminated in the marine ecosystem. Pigmented compounds from marine archaea are also prioritized owing to their ability to tolerate hyper saline and basic pH environments, besides their potential to withstand osmolytes (such as 2-sulfotrehalose) or high ionic strength [19,20].

Concerns regarding environmental conservation and consumers’ preferences have stimulated the interests of researchers and stake holders in exploring nontoxic, eco-friendly, and biodegradable commodities. Bacterially produced bio-pigments (bpBPs) have growing importance not only on account of their dyeing potential, but also due to their medicinal properties. Likewise, awareness regarding the carcinogenic and other pernicious effects of synthetic colorants has kindled a fresh enthusiasm towards the utilization of bacterial pigments in the food industry as safer alternatives to use as antioxidants, color intensifiers, flavor enhancers, and food additives.

Extraction of natural pigments from microorganisms populating environments exclusive of soil is a topic of current interest. Marine environment has become a captivating subject matter for microbiologists, pharmacologists, and biochemists in order to extract water based bacterial pigments. With the recent increase in awareness towards the benefits of natural over synthetic products, the bio-pigment industry is likely to increase its global market. The review aims at discussing the therapeutic and industrial significance of marine derived bacterial pigments helping to delineate the consequence of furthering the scope of these studies. It provides a comprehensive overview of potentiality and competence of marine bacteria as a source of bio-pigments by critically summarizing the scientific researches and accumulated data in the literature and the prominence of these bio-pigments in strengthening the overall pigment market by reviewing latest industry market research, reports, and statistics.

## 2. Marine Bacterial Species as Sources of Bio-Pigments

**Figure 1:**
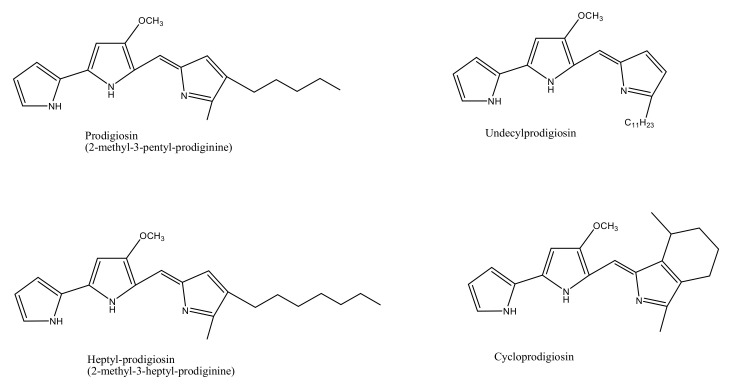

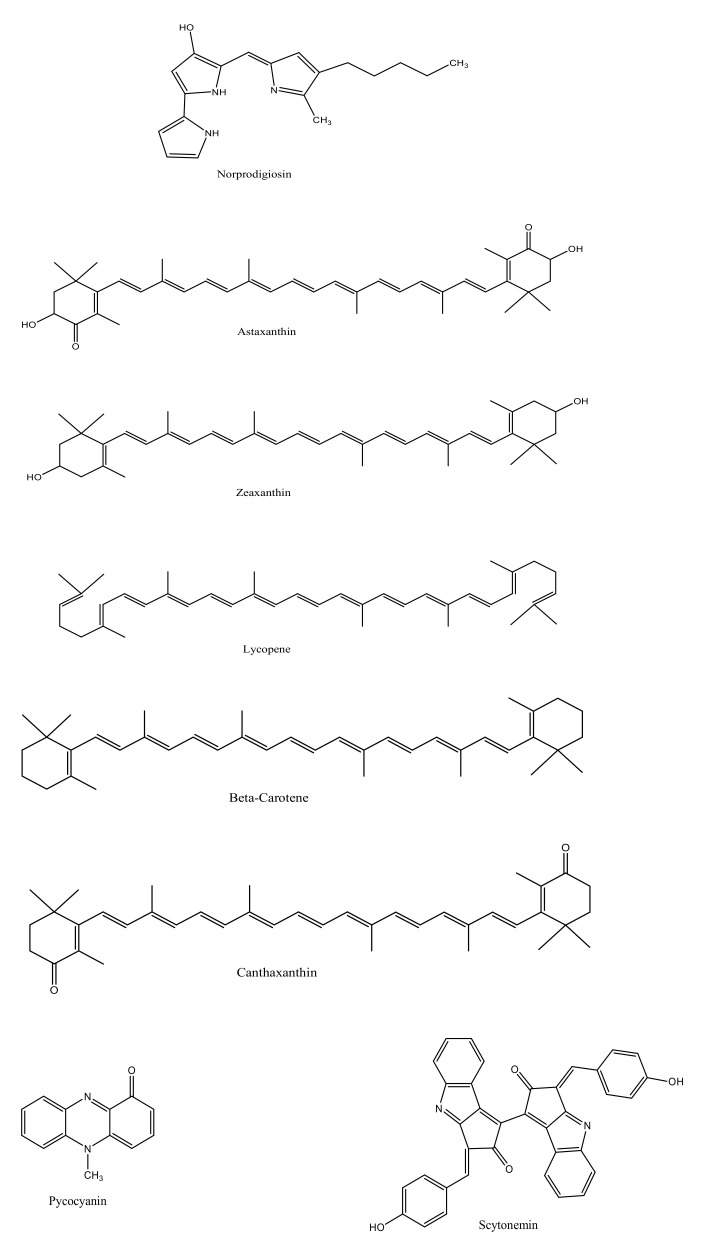

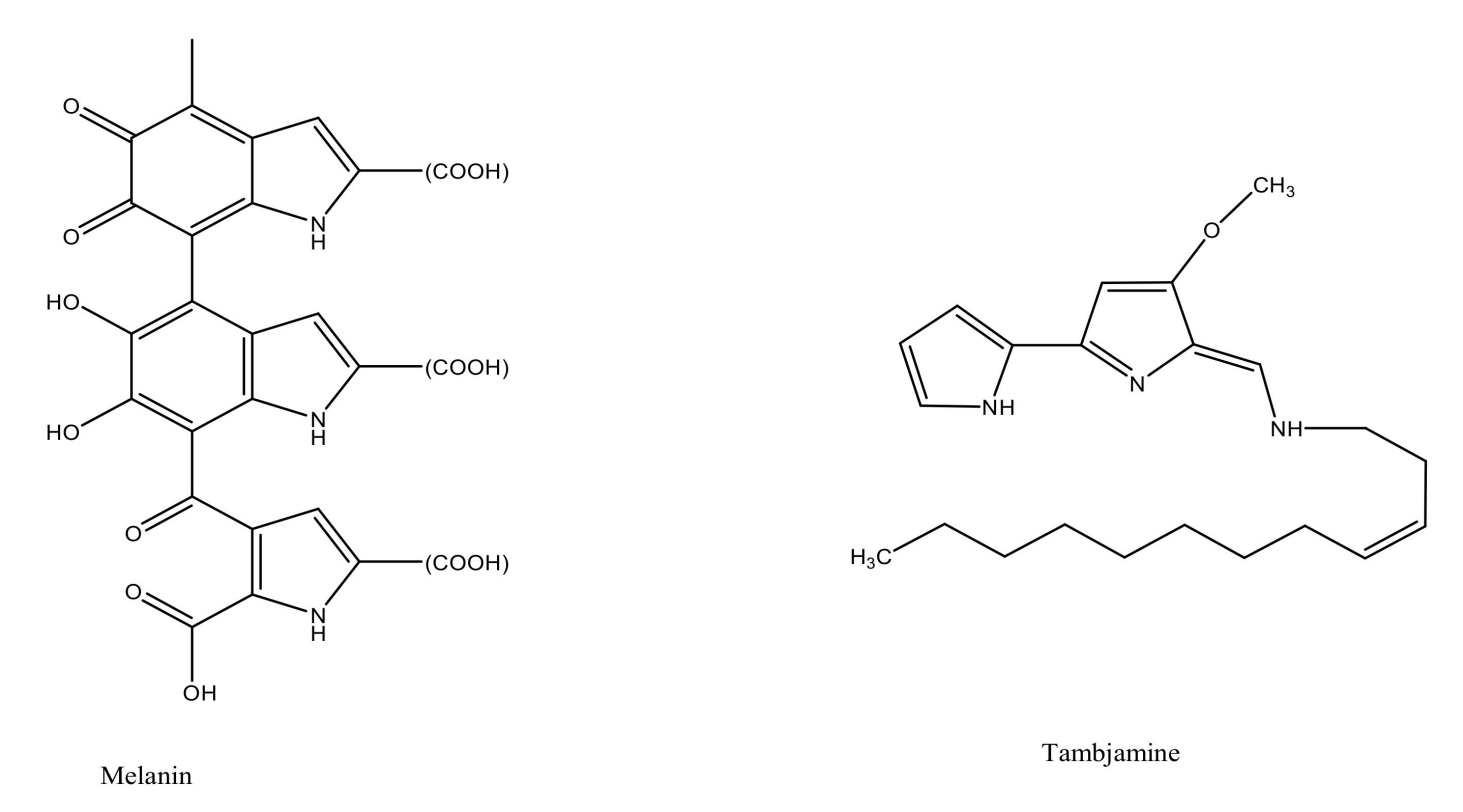
Chemical structures of various bacterial pigments.

The marine environment has been investigated for almost 300,000 known species, which constitutes only a small fraction of the total number of explorable pigment producing bacterial species. Bacterial species isolated from marine sediments or seawater such as *Streptomyces* sp., *Pontibacter korlensis* sp., *Pseudomonas* sp., *Bacillus* sp., and *Vibrio* sp. produce an array of pigmented compounds including prodigiosin, astaxanthin, pyocyanin, melanin, and beta carotene, respectively (Table 1). These pigments belong to a range of compound classes, for instance, carotenes are a subclass of carotenoids that have unsaturated polyhydrocarbon structures, prodiginines have a pyrrolyldipyrromethene core structure, tambjamines are alkaloid molecules, while violacein compounds are indole derivatives derived from tryptophan metabolism (Figure 1) [1,2,21]. These and other such pigments, despite their class diversity, share a functional likeness due to the presence of aromatic rings in their structures.

## 3. Biosynthesis of Bacterial Pigments

**Figure 2:**
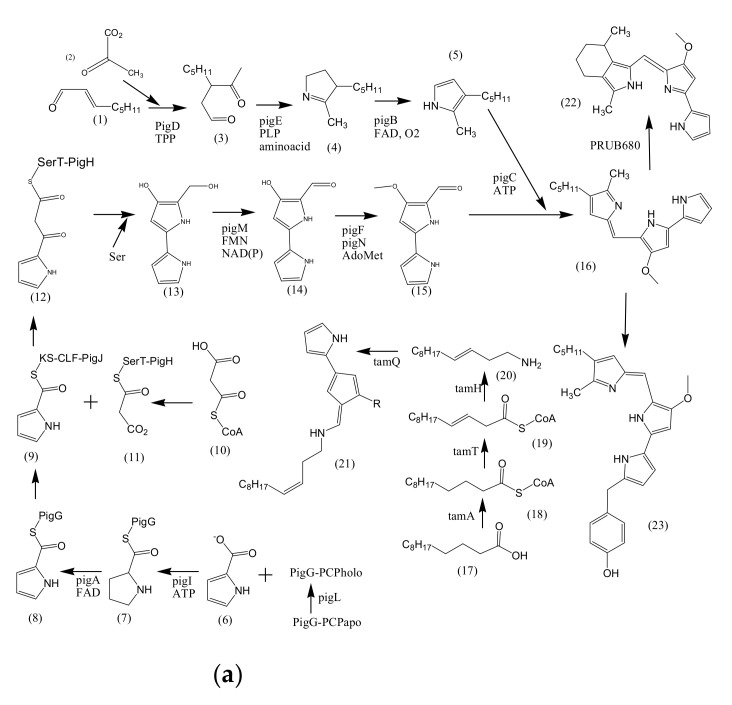

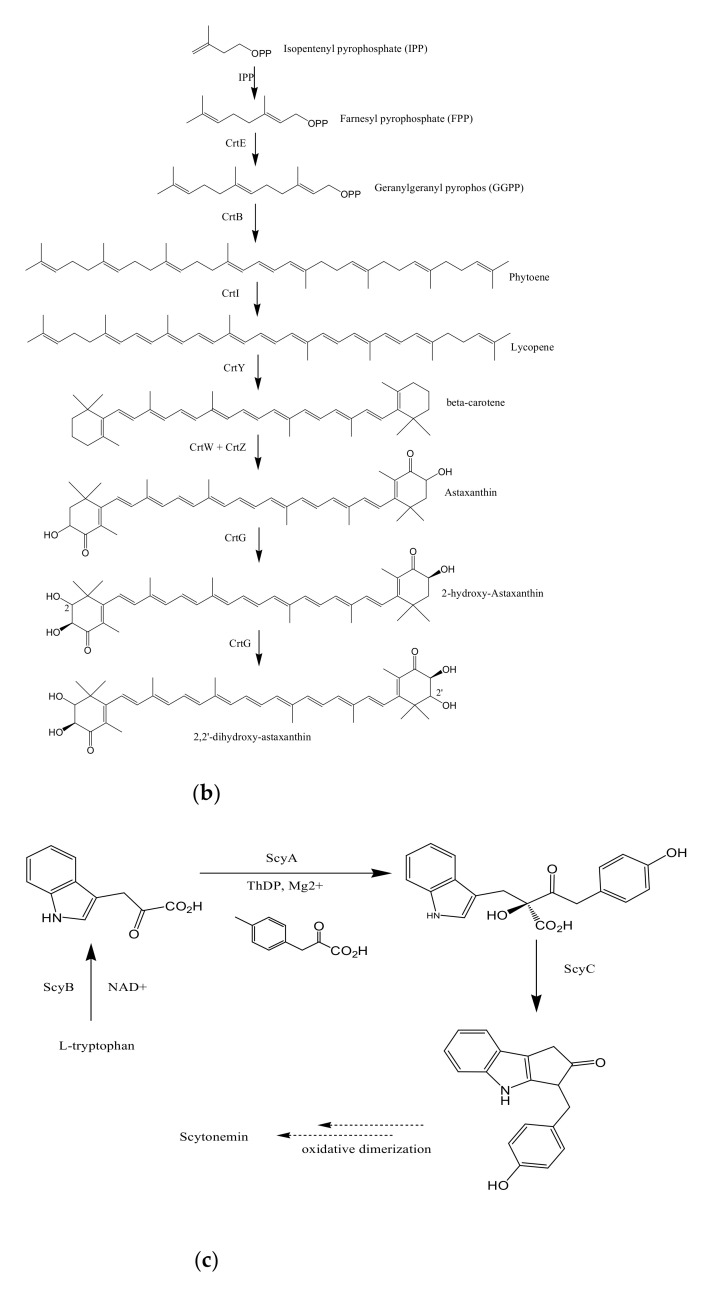

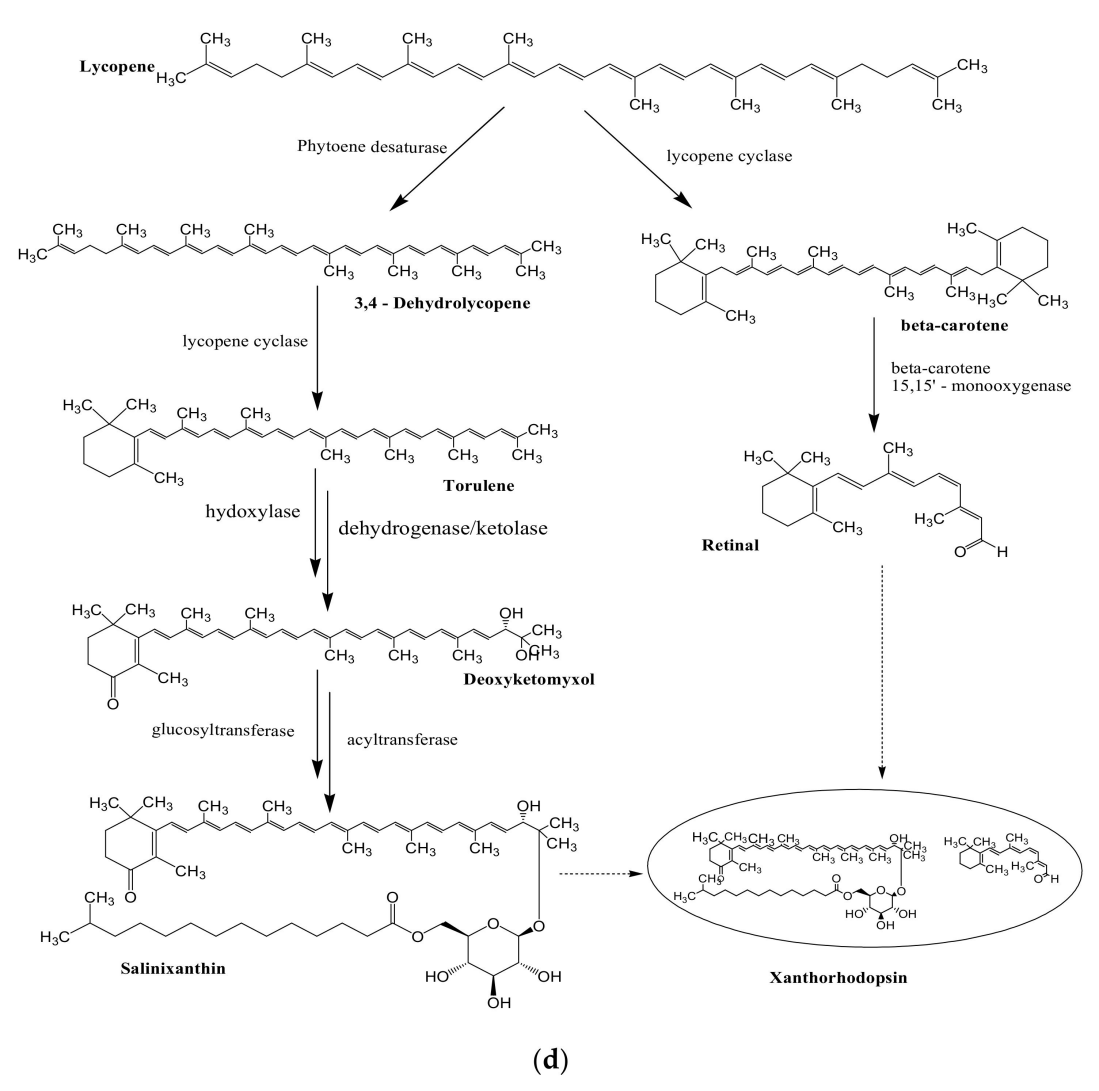
Proposed biosynthetic pathways of few bacterially produced bio-pigments. (**a**) Biosynthesis of Prodiginine analogs; MAP biosynthesis; MBC biosynthesis; Tambjamine biosynthesis; Cyloprodigiosin biosynthesis; 2-(p-hydroxybenzyl)prodigiosin (HBPG) biosynthesis. (**b**) Biosynthesis of carotenoids. (**c**) Biosynthesis of scytonemin. (**d**) Biosynthesis of salinixanthin and retinal pigments. (**a**) Biosynthesis of prodigioinine analogs. MAP Biosythesis (Green): (1) 2octenal, (2) Pyruvate, (3) 3-acetyloctanal, (4) H2MAP, (5) MAP. MBC Biosynthesis (Blue), (6) L-proline, (7) L-prolyl-S-PCP intermediate, (8) Pyrrolyl2-carboxyl-S-PCP, (9) Pyrrole-2-carboxyl thioester, (10) Malonyl-CoA, (11) Bound malonyl, (12) pyrrolyl-β-ketothioester on PigH, (13) 4-hydroxy-2,20-bipyrrole-5methanol (HBM), (14) 4-hydroxy-2,20-bipyrrole-5-carbaldehyde (HBC), (15) MBC, (16) Prodigiosin. Tambjamine Biosynthesis, (17) Dodecenoic acid, (18) Activated fatty acid, (19) CoA-ester, (20) Enamine, (21) Tambjamine, (22) Cycloprodigiosin (cPrG) &, (23) 2-(p-hydroxybenzyl)prodigiosin(HBPG) Biosynthesis. (**b**). Biosynthesis of carotenoids: CrtE: GGPP synthase, IPP: Isopentenyl pyrophosphate, GGPP: Geranylgeranyl pyrophos, CrtB: Phytoene synthase, CrtI: Phytoene desaturase, CrtY: lycopene β-cyclase, CrtW: β-carotene ketolase, CrtZ: β-carotene hydroxylase, CrtG: Astaxanthin 2,2′-β-ionone ring hydroxylase gene. (**c**). Biosynthesis of scytonemin: Scytonemin biosynthetic enzymes: ScyA, ScyB, ScyC (ScyA: a thiamin-dependent enzyme, ScyC: enzyme annotated as a hypothetical protein), ThDP: Thiamine diphosphate, NAD: Nicotinamide adenine dinucleotide, Mg^2+^: Magnesium ion.

The potential of marine bacterial isolates as a leading source of bio-pigments demands an extensive understanding of bio-mechanisms responsible for yielding pigmented molecules. Different studies have reported the proposed biosynthetic pathways of pigment production by marine bacterial isolates along with biochemically characterized enzymatic transformations (Figure 2). However, it is still unclear if the proposed pathways are distinct for marine or terrestrial bacterial species, or may be the same in both cases.

### 3.1. Biosynthesis of Prodiginine Analogs

2-methyl-3-n-amyl-pyrrole (MAP) biosynthesis: This pathway involves three genes; *pigB, pigD,* and *pigE*. At first, PigD carries out the addition of pyruvate to 2-octenal in the presence of coenzyme thiamine pyrophosphate (TPP). As a result, 3-acetyloctanal formation occurs along with the release of CO_2_ molecule. PigE catalyzes the transfer of an amino group to the aldehyde, followed by cyclization, resulting in the formation of H2MAP. PigB carries out further oxidation to form MAP (Figure 2a) [80,81].

4-methoxy-2,2′-bipyrrole-5-carbaldehyde (MBC) biosynthesis: This pathway involves seven genes: *pigA, pigF-J, pigL,* and *pigM*. 4′-phosphopantetheinyl transferase (PigL) carries out the activation of peptidyl carrier protein (PCP) domain of PigG by introducing 4′-phosphopantetheinyl group. Formation of L-prolyl-S-PCP intermediate occurs by the transfer of L-prolyl group of L-proline to the thiol group of phosphopantetheine, carried out by PigI and ATP. PigA further catalyzes the oxidation of the intermediate to pyrrolyl-2-carboxyl-S-PCP. Pyrrole-2-carboxyl thioester is generated by the transfer of pyrrole-2-carboxyl group of PigG to the cysteine active site at PigJ. Phosphopantetheinylated ACP domains of PigH provide binding sites for malonyl group of malonyl-CoA. Decarboxylation of bound malonyl results in condensation with pyrrole-2-carboxyl thioester and leads to the formation of pyrrolyl-β-ketothioester on PigH. Generation of 4-hydroxy-2,2′-bipyrrole-5-methanol (HBM) occurs by decarboxylation between serine and pyrrolyl-β-ketothioester, catalyzed by PigH [80,82]. 4-hydroxy-2,2′-bipyrrole-5-carbaldehyde (HBC) is formed when PigM oxidizes the alcohol group of HBM. Methyltransferase (PigF) and oxidoreductase (PigN) further carries out the methylation of HBC hydroxyl group to form MBC [81]. After the formation of MAP and MBC, PigC utilizes ATP to perform terminal condensation of these pyrroles, synthesizing prodigiosin.

Cycloprodigiosin (cPrG) biosynthesis: The cyclization of undecylprodiginine in order to form metacycloprodigiosin and butyl-meta-cycloheptylprodiginine is carried out by *mcpG* and *redG*, respectively [83]. Studies also revealed that a homologus gene (PRUB680) encodes an alkylglycerol monooxygenase-like protein away from *pig* biosynthetic gene cluster [84]. The respective enzyme demonstrates regiospecificity through C-H activation, resulting in cyclization of prodigiosin to form cPrG [78].

Tambjamine (tam) biosynthesis: Tambjamines have MBC moiety but lack MAP moiety, rather have an enamine group. Enamine biosynthetic pathway involves three genes; *tamT, tamH,* and *afaA* [85]. Acyl CoA synthetase (TamA) activates dodecenoic acid [86]. Dehydrogenase (TamT) carries out the oxidation of activated fatty acid, incorporating a π-bond to the fatty acyl side chain at its C-3 carbon. Further, the reduction of CoA-ester, followed by transamination to dodec-3-en-1-amine is facilitated by reductase/aminotransferase (TamH). MBC and enamine then undergoes condensation in order to form tambjamine, catalyzed by TamQ [85].

### 3.2. Biosynthesis of Carotenoids

Carotenoids are yellow, orange, and red colored pigmented compounds that are further subdivided into carotenes and xanthophylls. So far, 700 carotenoids have been reported, and among them beta-carotene, lutein, canthaxanthin, astaxanthin, lycopene, and zeaxanthin are the highly valued carotenoids [87]. Universal precursors for C40 and C50 carotenoid biosynthesis are two 5 C subunits: isopentenyl diphosphate (IPP) plus its isomeric form dimethylallyl diphosphate (DMAPP). IPP/DMAPP isomerase (IDI) carries out the isomerization of IPP into DMAPP. Geranylgeranyl diphosphate (GGPP) synthase further catalyzes the addition of one DMAPP molecule with three IPP molecules to generate an immediate precursor, i.e., C20 geranylgeranyl diphosphate (GGPP) [88]. Phytoene synthase carries out the first committed step of carotenoid biosynthesis i.e., condensing two GGPP molecules to form phytoene (C40), which is further desaturated by phytoene desaturase by the incorporation of four double bonds in its structure. This desaturated structure is a red-colored compound unlike its colorless parent molecule, and is called lycopene. Lycopene further undergoes several modifications to produce different carotenoids. Beta-carotene is generated by the cyclization of lycopene, carried out by lycopene beta-cyclase. It is then converted into canthaxanthin and zeaxanthin by catalytic activity of two protein classes: beta-carotene ketolase and beta-carotene hydroxylase, respectively, next to the formation of astaxanthin [30]. Beta-carotene ketolase represented by CrtW and CrtO types adds the ketone group to carbon 4/40 of the b-ionone ring. However, beta-carotene hydroxylase, encompassed by CrtR, CrtZ, and P450 types carries out the hydroxylation of carbon 3/30 of the b-ionone ring [89]. 2,2′-β-ionone ring hydroxylase introduces hydroxyl group to the β-ionone ring of astaxanthin and results in the formation of 2,2′-dihydroxy-astaxanthin (Figure 2b) [33].

### 3.3. Biosynthesis of Scytonemin

Biosynthesis of scytonemin involves three scytonemin biosynthetic enzymes; ScyA, ScyB, and ScyC. ScyB carries out the conversion of L-tryptophan to 3-indole pyruvic acid. ScyA (thiamin-dependent enzyme) performs the coupling of 3-indole pyruvic acid with p-hydroxyphenylpyruvic acid and results in the formation of b-ketoacid, whose cyclization is further carried out by ScyC (enzyme annotated as hypothetical protein) [90]. The resulting tricyclic ketone resembles half of the skeleton of scytonemin (Figure 2c) [91].

### 3.4. Biosynthesis of Salinixanthin and Retinal

Retinal: Lycopene cyclase converts lycopene into β-carotene. Breakdown of β-carotene into two retinal molecules is further catalyzed by a gene annotated as β-carotene 15,15′-monooxygenase (orf4) (Figure 2d) [92,93].

Salinixanthin: Xanthorhodopsin (orf2) (a light-driven proton pump) has two chromophores; retinal and salinixanthin [94,95]. Phytoene desaturase (CrtI) converts lycopene to 3,4-dehydrolycopene, which is further converted to torulene by lycopene cyclase [92]. Subsequently, conversion of torulene to salinixanthin is catalyzed by hydroxylase, ketolase or dehydrogenase, glucosyltransferase, and acyltransferase, having reactions involved similar to that of biosynthetic reactions of myxol and canthaxanthin [96,97].

## 4. Industrial and Therapeutic Applications

### 4.1. Therapeutic Applications

#### 4.1.1. Antibacterial Activity

Antibacterial properties of various bacterially produced bio-pigments of marine origin have been reported against an array of bacterial species, e.g., prodigiosin, cycloprodiogisin (from *Z. rubidus* sp. S1-1), and the yellow pigment (extracted from *Micrococcus* sp. strain MP76) have shown antibacterial activity against *Staphylococcus aureus* sp. and *Escherichia coli* sp. [98,99]. Other bacterial strains that are reportedly inhibited by prodigiosin and cycloprodigiosin are *Bacillus subtilis* sp. and *Salmonella enterica* serovar Typhimurium [98]. Likewise, the yellow pigment has shown activity against *P. aeruginosa* sp. as well [99]. Norprodigiosin synthesized by marine *Serratia* sp. has also been reported to exhibit inhibition activity against *Vibrio paraheamolyticus* sp. and *B. subtilis* sp. [32]. These studies strengthen the utilization of bpBPs as potential alternatives to synthetic medicinal compounds.

Furthermore, inhibition activities recorded against *Citrobacter* sp. by pyocyanin and pyorubin [58] and *P. aeruginosa* sp. by violacein pigment (purified from Antarctic *Iodobacter* sp.) [100], further stretches the range of marine-derived bpBP’s potential against pathogenic bacterial species to opportunistic bacterial species. There are numerous correspondingly published studies. The pigment “melanin” from marine *Streptomyces* sp., for instance, demonstrated antibacterial activity against *E. coli* sp., *S. typhi* sp., *S. paratyphi* sp., *Proteus mirabilis* sp., *Vibrio cholera* sp., *S. aureus* sp., and *Klebsiella oxytoca* sp. [68]. A bright pink-orange colored pigment extracted from *Salinicoccus* sp. (isolated from Nellore sea coast) also showed antimicrobial potential against several bacterial strains including *E.coli* sp., *Klebsiella pneumoniae* sp., *B. subtilis* sp., *Proteus vulgaris* sp., *P. aeruginosa* sp., and *S. aureus* sp. [101]. Hence, these and similar other studies all indicate the exploration of marine bacterial species as a dynamic approach to derive antibacterial compounds.

A few studies also seemingly suggest that a single pigment from different species may exhibit activities against various target microorganisms. One example is violacein, a violet colored pigment extracted from Antarctic bacterium *Janthinobacterium* sp. SMN 33.6, which showed antibacterial activity against multi-resistant bacteria: *S. aureus* sp. ATCC 25923, *E. coli* sp. ATCC 25922, *Kocuria rhyzophila* sp. ATCC 9341, and *S. typhimurium* sp. ATCC 14028 [102], and that extracted from *Collimonas* sp. showed antibacterial activity against *Micrococcus luteus* sp. [67].

#### 4.1.2. Antifungal Activity

Studies have also been carried out to determine the antifungal potential of natural pigmented compounds. Several studies have reported the antifungal activity of marine-derived bacterial pigments, among which violacein from *Chromobacterium* sp. and prodiginine pigments (prodigiosin and cycloprodigiosin) extracted from Indonesian marine bacterium *P. rubra* sp. reported to exhibit antagonistic activity against *Candida albicans* sp. [23,103]. Violacein also inhibited several other fungal strains, including *Penicillium expansum* sp., *Fusarium oxysporum* sp., *Rhizoctonia solani* sp., and *Aspergillus flavus* sp. Studies have also reported that violacein (extracted from a pure *Chromobacterium* sp.) shows comparable antifungal activity to that of bavistin and amphotericin B, highlighting the potential of marine-derived bpBPs as effective antifungal agents over existing synthetic antifungal compounds [103].

#### 4.1.3. Anticancer Activity

Exploring anticancer compounds from marine microbes has been considered a hot spot in natural product research. Several studies have been carried out in order to examine the antitumor ability of marine bacterial pigments. Anticancer activity of marine-derived bpBPs has been explored against several cancerous cell lines. Astaxanthin and 2-(p-hydroxybenzyl) prodigiosin (HBPG) isolated from *P. kolensis* sp. and *P. rubra* sp. displayed significant cytotoxicity against human breast cancer cell line (MCF-7) and human ovarian adenocarcinoma cell line, respectively [38,104]. PCA (Phenazine -1-carboxylic acid) pigment extracted from marine *P. aeruginosa* sp. GS-33 correspondingly showed inhibition against SK-MEL-2 (human skin melanoma cell line) [105]. Another pigment violacein extracted from Antarctic bacterium isolate, identified as a member of the genus *Janthinobacterium* (named as *Janthinobacterium* sp. strain UV13), revealed its antiproliferative activity in HeLa cells. Studies further confirmed the potential of violacein as an anticancer agent to cisplatin drug (anticancer chemotherapy drug) in cervix cell carcinoma [106]. It has also been reported that a single pigment can express anticancer activity against multiple cancerous cell lines. Synthetically derived tambjamines isolated from the marine bacterium *P. tunicata* sp. have shown significant apoptosis inducing effects against various cancer cell lines including glioblastoma cell line (SF-295), M14 melanoma cell line (MDA-MB-435), ileocecal colorectal adenocarcinoma cell line (HCT-8), and promyelocytic leukemia cells (HL-60) [107]. Carotenoid pigments extracted from marine *Arthrobacter* sp. G2O (isolated from the Caspian Sea) exhibited antitumor activity on esophageal squamous cancerous cells [108]. Likewise, prodigiosin homolog extracted from marine bacterium *Serratia proteamacula* sp. was also found to exhibit high antitumor activity [109], indicating the potential of marine-derived bpBPs in antitumor therapy.

#### 4.1.4. Antioxidant Activity

Marine-derived bpBPs are also being explored for their antioxidant activity. 3R saproxanthin and myxol pigments (from marine bacterium belonging to genus *Flavobacteriacae*) exhibited antioxidant activity against lipid peroxidation and also showed neuroprotective activity against L-glutamate toxicity [110]. The antioxidant activities of zeaxanthin (extracted from marine bacterium of genus *Muricauda*) [111] and melanin (from marine *Pseudomonas stutzeri* sp.) [112] have also been identified. Another pigment, phycocyanin extracted from marine bacterium *Geitlerinema* sp TRV57, demonstrated appreciable antioxidant activity [113]. Crude pigment extracted from the marine bacterium *Streptomyces bellus* sp. MSA1 also displayed 82% of DPPH (2,2-diphenyl-1-picryl-hydrazyl-hydrate) activity and said to exhibit radical scavenging activity [114]. Likewise, pigment crude extract from *Zobellia laminarie* sp. 465 (isolated from sea sponge) reported to exhibit high antioxidant values for ABTS-L (capture of the 2,2-azino-bis(3-ethylbenzothiazoline)-6-sulphonic acid (ABTS^+^) radical of the lipophilic fraction) [115], suggesting the importance of marine derived bacterial pigments in pharmaceutical and medicinal industries.

#### 4.1.5. Antiviral Activity

The advancing viral pandemics have taken a toll over the limited pool of existing antiviral agents, which has led to a rigorous search for newer, natural compounds with better antiviral capacities. Various studies on marine bpBPs suggest them as potential candidates. Prodigiosin extracted from *Serratia rubidaea* sp. *RAM_Alex* showed antiviral activity against hepatitis C virus (HCV) upon injecting HepG2 (human liver cancer cell line) cells with 2% of HCV infected serum (Table 2) [116]. Other carotenoid pigments (from *Natrialba* sp. M6) have also displayed complete elimination of HCV and clearance of 89.42% of hepatitis B virus (HBV) [117], indicating the use of marine pigments as availing antiviral agents.

### 4.2. Industrial Applications

#### 4.2.1. Bio-Pigments as Food Colorants

Researchers have concluded that marine-derived bpBPs can be utilized to provide full-scale commercial production of food-grade pigments, owing to their little or no threats to consumer health. They also showed pleasant colors at low concentrations. Pyorubrin and pyocyanin, for example, extracted from *P. aeruginoasa* sp., when assessed for their utilization as food colorings with agar, gave pleasing colors at 25 mg mLG^−1^ [58]. The utilization of bpBPs was also suggested as a feed additive to promote growth and enhance the coloration of ornamental fishes [119]. Furthermore, prodigiosin (from marine bacterium *Zooshikella* sp.) has been reported to exhibit good staining properties and a three months shelf life [120], which hints toward a sustainable aspect of marine-derived pigmented molecules as food colorants.

#### 4.2.2. Bio-Pigments as Dyeing Agents

The worldwide demand for clothes is rising exponentially. Newly, there is an increase in the insistence of incorporating antimicrobial properties in fabrics. Lee et al. identified a novel marine bacterium *Z. rubidus* sp. S1-1 that produced two significant pigments, i.e., prodigiosin and cycloprodigiosin. These were used to dye cotton and silk fabrics. Results revealed that the application of red-pigmented extract solution on fabrics reduced the growth rate of *S. aureus* sp. KCTC 1916 by 96.62% to 99.98% and *E. coli* sp. KCTC 1924 by 91.37% to 96.98% [98]. Furthermore, *Vibrio* sp. isolated from marine sediment produced a bright red colored prodiginine pigment that was used to dye nylon 66, silk, wool, acrylic, and modacrylic fabrics to obtain a pretty deep-colored shade. The dyed silk and wool fabrics also showed antibacterial activity against *E. coli* sp. and *S. aureus* sp. [121]. Researchers at Ulsan National Institute have also reported the synthesis of antibacterial fabric by using violacein pigment extracted from *C. violacea* sp. [122,123]. Prodigiosin pigment extracted from *Serratia* sp. BTWJ8 effectively dyed paper, PMMA (Polymethyl methacrylate sheets), and rubber latex. Rubber is commonly used in day to day life either in houses or industries. PMMA have been widely utilized for the construction of lenses for exterior lights of automobiles. Different concentrations of prodigiosin produced variable color shades that revealed its affectivity as a coloring agent [124].

#### 4.2.3. Use in Cosmetics

The cosmetic industry is an expeditiously emerging global business market. About 2000 companies in the United States of America are cosmetic manufacturers. It is estimated that American adults use seven different skincare products per day for everyday grooming [125]. The cosmetic industry has a worth of 10.4, 10.6, and 13.01 billion euros in the UK, France, and Germany, respectively [126]. Considering the cosmetic market value worldwide, researchers have also made efforts to explore the use of marine-derived bpBPs in skincare products. The addition of the pigment PCA in a solution of commercial sunscreen enhanced its UV-B (ultraviolet B-rays) protection and increased the SPF (sun protection factor) values up to 10% to 30% [105].

Similarly, melanin incorporated cream (named cream F3) was synthesized by concentrates of seaweed (*Gelidium spinosum*) and melanin pigment (extracted from marine bacterium *Halomonas venusta* sp.). Cream F3 showed high SPF values and photoprotective activity and demonstrated great effectivity in wound healing as well. Moreover, the formulated cream also exhibited antibacterial activity against skin pathogens; *Streptococcus pyogenes* sp. (MTCC 442), and *S. aureus* sp. (MTCC 96) [127]. Another research reported the effectivity of melanin (extracted from marine bacterium *Vibrio natriegens* sp.) in protecting mammalian cells from UV irradiation. Results revealed 90% survival rate of HeLa cells in melanized cell culture [128]. In another report, Bio lip balm made from crude pigment (extracted from *S. bellus* sp. MSA1) in a mixture of coconut oil, lanolin, and shredded bee wax [114] suggested the use of melanin pigment as a significant ingredient in several beauty care products as well.

#### 4.2.4. Antifouling Agent

Billions of dollars have been spent each year to control fouling activities on different objects placed in the marine environment. Biofouling on ships such as dreadnoughts increased the roughness of the hull, which promotes frictional resistance, ultimately leading to an increase in fuel consumption and other corresponding environmental compliances. Heavy metal-based antifoulants cause severe environmental complications, which further mandate the need for “eco-friendly” antifouling agents. Researchers have also revealed the use of marine-derived bpBPs for their role as an antifouling agent, for instance, prodigiosin extracted from *Serratia.* sp. was reported to exhibit antifouling activity against marine fouling bacterial species such as *Gallionella* sp. and *Alteromonas* sp. It also inhibited the adhesion of *Cyanobacterium* sp. on the glass surface [129]. Likewise, another pigment, polymelanin synthesized by the marine bacterium *P. lipolytica* sp., prevented metamorphosis and decreased the invertebrate larval settlement [130], hence indicating the role of marine bacterial pigments as potential antifoulants.

#### 4.2.5. Photosensitizers

The use of prodigiosin has also been reported as photosensitizers in solar cells. The high photostability of extracted prodigiosin demonstrated its use as a sensitizer in dye-sensitized solar cells (DSSC) (Table 3) [131]. This study suggests the viability of bpBPs in addition to that of prodigiosin for the construction of cost-effective and low tech industrially produced DSSC.

## 5. Industrial Importance and Global Market Trends of Pigmented Compounds

**Figure 3:**
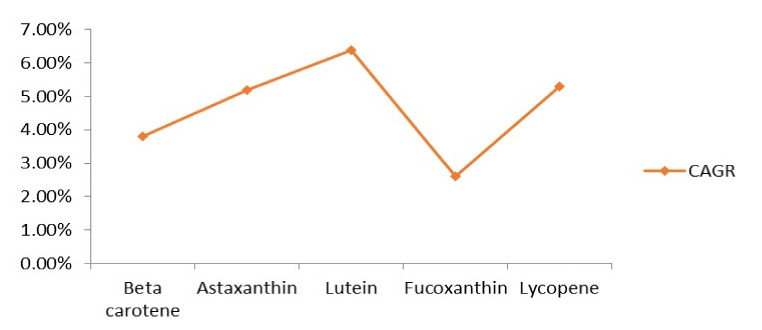
Prospective compound annual growth rate (CAGR) of several pigments by the year 2026 [161,162,163,164,165].

Pigments are already utilized in various nutritional supplements, antibiotics, skin care, and other industrial products (Table 4). The most valuable pigments in the global market are beta-carotene, lutein, and astaxanthin (Figure 3). Astaxanthin has its wide use in nutraceutical industries owing to its antioxidant properties and numerous health benefits. It has also been in wide use in cosmetic industries due to its antiaging activity. Moreover, astaxanthin is being utilized in aquaculture industries to carry out the pigmentation of shrimps, trouts, and salmons. At the industrial scale, astaxanthin production is accomplished using *Paracoccus* sp. It was predicted that the sales volume of astaxanthin by the year 2020 would be 1.1 billion US dollars [132], and the astaxanthin market is estimated to reach up to 3.4 billion US dollars with CAGR (compound annual growth rate) of 16.2% in 2027 [133].

FDA accepted the use of beta-carotene as a color additive in food products in the year 1964. Additionally, in 1977, the use of beta-carotene got approved in cosmetics also. The E-Number allotted to beta-carotene is E160a. Over and above, canthaxanthin use in food and broiler chicken feed got authorized in 1969, and the E-Number assigned to it is E161g [159]. Lycopene is also being utilized for many industrial purposes, and it is reckoned that the lycopene market will grow at a rate of 5.3% CAGR, by the end of 2026 [160].

**Figure 4:**
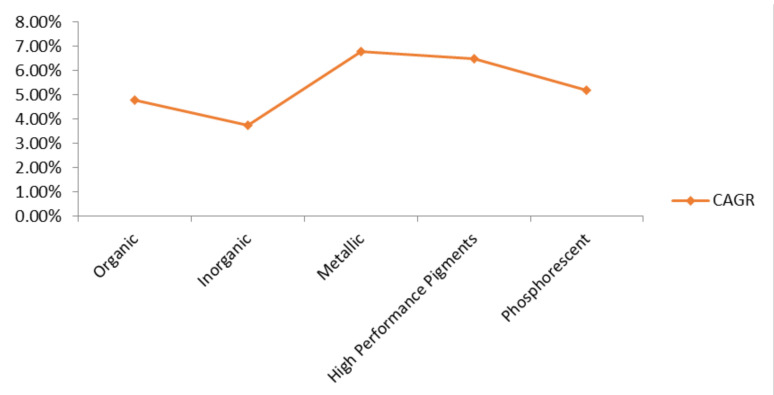
Expected market worth of different classes of pigments during, the forecasting period: 2019 to 2026 [169,170,171,172,173].

The global market potential of carotenoid pigments is estimated to reach up to 5.7% by 2022 [166]. Europe and USA are the key business markets for carotenoid pigments [118]. It is expected that the global carotenoid market will increase from 1397.59 million US$ in 2018 to 2124.68 million dollars by the end of 2025, at the CAGR of 6.16% [167]. For a long time, different classes of pigmented compounds have occupied the entire market due to their wide range of applications in different industries (Figure 4). It is anticipated that by the year 2022, the global market of food colorants will reach up to 3.75 billion US$, along with farming colorant market, to touch 2.03 billion US$ by the year 2022 [166]. Europe holds the forefront for cutting synthetic colorants’ economy by utilizing natural dyes, which make up 85% of total dyes produced. It is evaluated that growing interests towards ready to eat and pre-packaged food items in China, India, and Middle-East countries will also drive the market of food colorants in the Asia Pacific as well [168].

## 6. Conclusions

Marine bacterial pigments can be a potential substitute for synthetic products to fulfill market demand and to ensure the public well-being. Putting aside the fact that synthetic medicines combat bacterial infections, they also pose adverse effects in terms of health. Likewise, artificial colorants due to the presence of azo dyes and heavy metals can also ignite cancer and other allergies. Microbial pigments derived from marine bacteria can be a promising approach to tackle the detrimental effects of these synthetic compounds.

Furthermore, marine bacteria tolerate a vast range of environmental conditions. Due to their unique biological properties, natural pigments from the marine environment also have wide range of applications in pharmaceutical, food, cosmetics, paper, and textile industries [1]. Marine bacterial species can be cultured in vitro and are genetically modified to get the desired level of pigments. Anyhow, there are certain limitations in implementing these naturally derived marine bacterial pigments on an industrial scale significantly: less percentage annual production, technological imperfections, low stability, high need for cost investments, and health complications [11]. For replacing synthetic products, efforts are required to explore new microbial sources and finding better optimization techniques to enhance the production of pigmented compounds. Genetic engineering and other strain development techniques should also be further studied to harvest bioactive pigments from marine bacterial species.

## Figures and Tables

**Table 1:** Marine bacterial sources of colored pigmented compounds.

Pigments	Marine Bacterial Species	References
Prodigiosin	*Hahella chejuensis* sp. *Pseudoalteromonas rubra* sp. *Streptomyces* sp. SCSIO 11594 *Vibrio* sp. (Strain MI-2) *Serratia marcescens* sp. IBRL USM 84 *Zooshikella ganghwensis* gen. nov., sp. nov.	[22,23,24,25,26,27]
Undecylprodigiosin	*Streptomyces* sp. UKMCC_PT15 *Streptomyces* sp.SCSIO 11594 Novel strain of Actinobacterium sp., *Saccharopolyspora* sp.	[24,28,29]
Heptylprodigiosin	*Spartinivicinus ruber* gen. nov., sp. nov	[30]
Cycloprodigiosin	*Pseudoalteromonas denitrificans* sp. *Pseudoalteromonas rubra* sp. ATCC 29570	[15,31]
Norprodigiosin	*Serratia* sp. WPRA3	[32]
Astaxanthin	*Brevundimonas scallop* sp. Zheng & Liu *Corynebacterium glutamicum* sp. *Brevundimonas* sp. strain N-5 *Sphingomicrobium astaxanthinifaciens* sp. nov *Rhodovulum sulfidophilum* sp. *Pontibacter korlensis* sp. AG6 *Exiguobacterium* sp. *Altererythrobacter ishigakiensis* sp. NBRC 107699 *Rhodotorula* sp. *Paracoccus haeundaensis* sp.	[33,34,35,36,37,38,39,40,41,42]
Zeaxanthin	*Sphingomonas phyllosphaerae* sp. KODA19-6 *Mesoflavibacter aestuarii* sp. nov. *Aquibacter zeaxanthinifaciens* gen. nov., sp. nov. *Zeaxanthinibacter enoshimensis* gen. nov., sp. nov *Gramella planctonica* sp. nov *Mesoflavibacter zeaxanthinifaciens* gen. nov., sp. nov *Formosa* sp. KMW *Sphingomonas phyllosphaerae* sp. FA2^T^ *Sphingomonas (Blastomonas) natatoria* sp. DSM 3183^T^ *Muricauda lutaonensis* sp. CC-HSB-11T	[29,43,44,45,46,47,48,49,50]
Lycopene	*Blastochloris tepida* sp. *Salinicoccus roseus* sp.	[51,52]
Beta carotene	Cyanobacterium *Synechococcus* sp. *Micrococcus* sp. *Vibrio owensii* sp. *Flavicella marina* gen. nov., sp. nov. *Gordonia terrae* sp.TWRH01	[53,54,55,56,57]
Canthaxanthin	*Brevibacterium* sp. *Gordonia* sp. *Dietzia* sp.	[54]
Pyocyanin	*Pseudomonas aeruginosa* sp.	[58]
Scytonemin	*Cyanobacterial* sp. *Nostoc punctiforme* sp. ATCC	[59,60]
Violacein	*Pseudoalteromonas luteoviolacea* sp. *Pseudoalteromonas ulvae* sp. TC14 *Pseudoalteromonas* sp. 520P1 *Microbulbifer* sp. D250 *Pseudoalteromonas amylolytica* sp. nov *Chromobacterium violaceum* sp. *Collimonas* sp.	[61,62,63,64,65,66,67]
Melanin	*Streptomyces* sp. *Pseudomonas* sp. *Marinomonas mediterranea* sp. MMB-1T *Pseudomonas stutzeri* sp. *Bacillus* sp. BTCZ31 *Streptomyces* sp. MVCS13 *Providencia rettgeri* sp. strain BTKKS1 *Marinobacter alkaliphilus* sp. *Leclercia* sp. *Halomonas meridian* sp. *Nocardiopsis dassonvillei* sp. strain JN1 *Vibrio alginolyticus* sp. strain BTKKS3	[68,69,70,71,72,73,74,75,76,77]
Tambjamines	*Pseudoalteromonas tunicata* sp. *Pseudoalteromonas citrea* sp.	[78,79]

**Table 2:** Therapeutic applications of bio-pigments extracted from marine bacterial isolates.

Pigments	Marine Bacterial Species	Therapeutic Applications	References
Prodigiosin and cycloprodigiosin	*Zooshikella rubidus* sp. S1-1	Antibacterial	[98]
	*Pseudoalteromonas rubra* sp. PS1 and SB14	Antifungal	[23]
Prodigiosin	*Serratia rubidaea* sp. *RAM_Alex*	Antiviral	[116]
2-(p-hydroxybenzyl) prodigiosin	*Psuedoalteromonas rubra* sp.	Cytotoxic	[104]
Astaxanthin	*Pontibacter korlensis* sp. AG6	Anticancer	[38]
2,2 dihydroxyastaxanthin	*Brevundimonas* sp.	Antioxidant	[33]
Zeaxanthin	*Muricauda aquimarina* sp. *Muricauda olearia* sp.	Nitric oxide scavenging Inhibition of lipid peroxidation DPPH radical scavenging activities	[111]
Lycopene	*Arthrobacter* sp. G2O	Antitumor	[108]
Beta Carotene	*Cyanobacterium* sp.	Antioxidant Antidiabetic Antitumor	[118]
Pyocyanin and pyorubrin	*Psuedomonas aeroginosa* sp.	Antibacterial	[58]
Melanin	*Streptomyces* sp.	Antibacterial	[68]
	*Nocardiopsis* sp.	Antiquorum sensing	[76]
	*Pseudomonas stutzeri* sp.	Antioxidant	[112]
Poly melanin	*Leclercia* sp. BTCZ22	Antibiotic resistance	[75]
Tambjamines	*Psuedoalteromonas tunicata* sp.	Anticancer	[107]
Violacein	*Janthinobacterium* sp. SMN 33.6	Antibacterial	[102]
	*Collimonas* sp.	Antibacterial	[67]
	*Iodobacter* sp.	Antibacterial	[100]
	*Chromobacterium* sp.	Antifungal	[103]
	*Janthinobacterium* sp. UV13	Anticancer	[106]
3R Saproxanthin and Myxol	*Flavobacteriacae* sp.	Antioxidant	[110]
PCA	*Pseudomonas aeruginosa* sp. GS-33	Effectivity against melanoma cell cancer	[105]
Bright pink-orange colored pigment	*Salinicoccus* sp.	Antibacterial	[101]
Yellow pigment	*Micrococcus* sp. MP76	Antibacterial	[99]

**Table 3:** Industrial applications of bio-pigments extracted from marine bacterial isolates.

Pigments	Marine Bacterial Species	Industrial Applications	References
Prodigiosin and cycloprodigiosin	*Zooshikella rubidus* sp. S1-1	Dyeing potential	[98]
Prodigiosin	*Vibrio* sp.	Dyeing of fabrics	[121]
	*Serratia marcescens* sp.11E	Photosensitizers	[131]
	*Serratia* sp. BTWJ8	Dyeing of paper, PMMA and rubber latex	[124]
	*Zooshikella* sp.	Food colorant Staining	[120]
	*Serratia marcescens* sp. CMST 07	Antifouling	[129]
Lycopene	*Streptomyces* sp.	Food grade pigments Feed additive Colorant	[119]
Pyocyanin and pyorubrin	*Psuedomonas aeroginosa* sp.	Food colorings	[58]
Scytonemin	*Lyngbya aestuarii* sp.	Sunscreen	[91]
Melanin	*Vibrio natriegens* sp.	Protection from UV irradiation	[128]
	*Halomonas venusta* sp.	Sunscreen Wound healing	[127]
	*Nocardiopsis* sp.	Antibiofilm	[76]
Poly melanin	*Pseudoalteromonas lipolytica* sp.	Antifouling agent	[130]
	*Vibrio natriegens* sp.	Removal of heavy metals and environmental pollutants	[128]
PCA	*Pseudomonas aeruginosa* sp. GS-33	Enhance SPF values UV-B protection	[105]

**Table 4:** Different industrial products and nutritional supplements utilizing pigmented compounds along with manufacturers, product brands, suppliers and company coverage.

Pigments	Products/Nutritional Supplements	Company Coverage/Manufacturers/ Product Brands/Suppliers	References/Links
Pigments from Bacterial Origin			
Prodigiosin	Prodigiosin *Serratia marcescens-*CAS 82-89-3-CalbiocheM	Sigma-Aldrich	[134]
Astaxanthin	*Paracoccus* Powder (Astaxanthin Powder) 2Z	Brine Shrimp Direct	[135]
	*Paracoccus* Powder, Natural Source of Astaxanthin, 50g	NoCoast AQUATICS	[136]
Violacein	Violacein (from *Janthinobacterium lividum*)	Sigma-Aldrich	[137]
Synthetic Pigments and Pigments derived from other sources			
Prodigiosin	Prodigiosin, Antibiotic Prodigiosin 25c	My BioSource Leap Chem Co., Ltd.	[138,139,140]
		Prodigiosin Suppliers	
		Hangzhou Dayangchem Co., Ltd. Puyer Bio Pharm Ltd Santa Cruz Biotechnology	
Astaxanthin	Lucantin^®^ Pink (Astaxanthin) AstaSana™ 10% FS J-Bio^TM^Astaxanthin BioAstin® Hawaiian Astaxanthin	BASF Nutrition DSM GMP Global Marketing, Inc. Cyanotech Corporation	[141,142,143,144,145]
		Astaxanthin Suppliers	
		Aecochem Corp. Simagchem Corporation Hangzhou Dayangchem Co., Ltd. Xiamen Hisunny Chemical Co., Ltd.	
Zeaxanthin	ZeaGold®, Zeaxanthin MacuShield® softgel capsule OPTISHARP® (Zeaxanthin) 20% FS.	Kalsec AGP Limited DSM Nutritional Products, Inc.	[146,147,148,149]
		Zeaxanthin Suppliers	
		Shanghai Worldyang Chemical Co., Ltd. Sancai Industry Co., Ltd. Carbone Scientific Co., Ltd. BLD Pharmatech Ltd.	
Lycopene	redivivo® (Lycopene) 10% FS Lyc-O-Mato®	DSM Nutritional Products, Inc. LycoRed Ltd.	[150,151,152]
		Lycopene Suppliers	
		Haihang Industry Co., Ltd. Shanghai Worldyang Chemical Co., Ltd. Junwee Chemical Co., Ltd. B.M.P. Bulk Medicines & Pharmaceuticals GmbH.	
Beta-Carotene	Beta-Carotene 10% DC Beta-Carotene 1% SD Beta-Carotene 30% in corn oil CaroCare®, Beta-Carotene Lyc-O-Beta 7.5% VBA	Barrington Nutritionals DSM Nutritional Products LycoRed Ltd.	[153,154,155,156]
		Beta-Carotene Suppliers	
		Puyer BioPharma Ltd. Sancai Industry Co., Ltd. United New Materials Technology SDN.BHD. Hangzhou Keying Chem Co., Ltd.	
Lutein	FloraGLO® Lutein 20% SAF	DSM Nutritional Products, Inc.	[157,158]
		Lutein Suppliers	
		Beckmann-Kenko GmbH. New Natural Biotechnology Co., Ltd. BuGuCh & Partners. Stauber Performance Ingredients, Inc. (previous Pharmline, Inc.)	
